# Concurrent Formation of Ice Network within Mineral Colloids with Suppressed Volume Expansion

**DOI:** 10.1021/acs.jpclett.5c03627

**Published:** 2026-01-19

**Authors:** Hongkun Li, Yunchen Long, Junda Shen, Xinxue Tang, Jiahua Liu, Chong Wang, Binbin Zhou, Bo Li, Jing Zhong, Xiao Ma, Chunyi Zhi, Jian Lu, Yang Yang Li

**Affiliations:** † Hong Kong Branch of National Precious Metals Material Engineering Research Center, City University of Hong Kong, 999077 Hong Kong SAR, China; ‡ Centre for Advanced Structural Materials, City University of Hong Kong Shenzhen Research Institute, Greater Bay Joint Division, Shenyang National Laboratory for Materials Science, Shenzhen 518057, China; § Department of Materials Science and Engineering, 597169 City University of Hong Kong, 999077 Hong Kong SAR, China; ∥ Department of Mechanical Engineering, City University of Hong Kong, 999077 Hong Kong SAR, China; ⊥ Department of Materials Science and Engineering, College of Transportation Engineering, Dalian Maritime University, Dalian 116026, PR China; # Shenzhen Institute of Advanced Electronic Materials, Shenzhen Institute of Advanced Technology, Chinese Academy of Sciences, Shenzhen 518055, China; 7 Department of History of Science and Scientific Archaeology, University of Science and Technology of China, Hefei 230026, China; 8 Department of Mechanical Engineering, The University of Hong Kong, 999077 Hong Kong SAR, China

## Abstract

The freezing behaviors of water are one of the most critical factors that define the formats of life and the landscapes on Earth. The current methods for regulating the freezing behaviors mainly rely on ice-structuring proteins or nanomaterials to hinder the conversion of water into crystalline ice (*I*_h_) under low temperatures. Here we report that the presence of minuscule mineral particles can significantly suppress the volume expansion of water upon freezing into ice. In particular, colloidal precipitates of calcite, a primary mineral accounting for ∼4 wt % of the Earth’s crust and the most abundant biomineral on Earth, are able to reduce water expansion by 69% (from 8.4% to 2.6%) at 243 K. The mechanism of expansion suppression involves the formation of a continuous network of fairly ordered “ice-like” hydration waters that are bound to the surface of the mineral colloids at room temperature, and their concurrent crystallization through heterogeneous nucleation upon freezing, which confines the interstitial free water and refrains its volume expansion. These findings reveal the remarkable ability of common minerals to suppress a most ubiquitous phenomenon, water volume expansion upon freezing, and offer fresh insights into various fields such as biomineralization, hydrology, soil science, and lithology.

## Graphical abstract

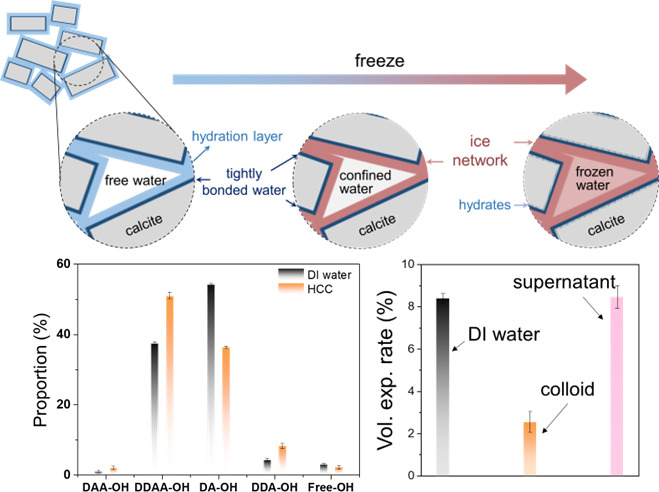

The unique freezing behaviors of water are essential in various fields, such as biology, geology, industry, and agriculture.^1–11^ The most interesting feature is the unusual volumetric expansion upon freezing into crystalline ice *I*_h_, which is not only critical for preserving aquatic life in cold climates but also a major cause of biological frostbite.^12–14^ The water molecule coordinates with 0 to 4 neighbors at the liquid state, whereas *I*_h_ ice consists of stacks of puckered hexagonal planes with each water molecule forming 4 hydrogen bonds (3 in the plane and 1 connecting the adjacent plane).^15^ The abnormal volume expansion upon freezing is a result of the formation of a more open lattice held up by a larger number of hydrogen bonds.^16^ Originally, ice-structuring proteins (ISP) rich in alanine, threonine, galactose, and N-acetyl galactosamine were believed to possess a high surface energy that can tightly bind to free water through hydrogen bonds and prevent them from turning into *I*_h_ ice, slowing down ice growth and enabling varied thermodynamic properties of ice.^1,17^ Similar regulatory effect on water freezing behavior was also discovered in polymer materials that are capable of strong bonding with water molecules.^18–21^ However, a detailed and systematic study of the ice-binding and nonice-binding faces of antifreezing proteins (AFP) provides a deep understanding of ice-structuring behavior. The nonice-binding face of AFP, due to its low compatibility with ice order, disrupts the hydrogen bonding network of water and thus becomes the core strength to inhibit the ice crystal growth, whereas the ice-binding faces facilitate the formation of the ice lattice.^22^

There are very few types of inorganic materials (mainly mesoporous silica and carbon nanomaterials) that are recognized as ice-structuring materials. For mesoporous silica, smaller pores were reported to more effectively promote the emergence of the less common ice phase *I*_c_, due to the larger content of interface water and the stronger confinement effect.^23^ Carbon-based materials have also attracted great attention in recent years for their influence on the freezing behaviors of water: Oxidized carbon nanomaterials were used to study the heterogeneous ice nucleation.^24^ Subsequently, through altering the size of graphene oxide nanosheets, the existence and the size range of critical ice nucleus during water freezing were experimentally demonstrated for the first time.^25^ Graphene oxide and oxidized quasi-carbon nitride quantum dots were shown to be good inhibitors for the growth or enlarged grain size of ice.^26,27^ Moreover, carbon nanotubes have a remarkable impact on the phase transformation of water confined within them: a single-walled carbon nanotube with a diameter of 1.05 nm can elevate the melting point of the ice trapped inside to even above 105 °C.^28^

Although researchers have made important discoveries in the freezing behaviors of water,^29,30^ there is a lack of effective ways to address the grand challenge of controlling the volume expansion of water upon freezing into *I*_h_. Here this study discloses that substantial suppression of the volume expansion of water upon freezing into ice (e.g., from 8.4% to 2.6% at 243 K) can be achieved via the minuscule particles of minerals, the most common and ubiquitous components of Earth’s crust, e.g., calcite which is a primary component of sedimentary rocks such as limestone and marble, comprises ∼4 wt % of the crust, and plays a significant role in biomineralization, geological processes and carbon cycling.^31,32^ Different from the previous studies which focus on the formation of ice within rocks and the counterforce provided by the rock structures or the capillary force against expansion during freezing-thawing cycles.^33^ The suppression of expansion via flowable mineral colloids is achieved through the formation of a hydrogen bonding network at the mineral surface at room temperature. These underscore the vital role of minerals as a key factor in understanding many important issues such as historical relics conservation, evolution of life, geomorphic changes, and soil formation.

As mentioned above, the extraordinary ice-regulating capabilities of organic macromolecules and inorganic nanomaterials are attributed to two key factors in literature: the strong interactions between water and substrates (e.g., the formation of surface hydration on ISP) and the spatial confinement of water (e.g., as in mesoporous silica or carbon nanotubes). In this context, closely packed mineral colloids represent a potentially significant class of undiscovered ice-structuring materials. The abundant metal or oxygen ions present on the surfaces of these colloids can form strong hydrogen bonds with adjacent water molecules, resulting in the creation of appreciable surface hydration layers. These layers may exhibit a lower density than free water due to their relatively ordered structure, leading to less expansion upon freezing. Furthermore, when mineral colloids are closely packed, the surface hydration layers come into contact with one another, forming a continuous hydration network throughout the colloidal system. Upon cooling, these hydration layers facilitate heterogeneous nucleation, enabling concurrent and rapid freezing to form a continuous nanostructured ice framework. This framework can exert a confinement effect on the interstitial free water trapped among the colloidal particles (the volumetric ratio of water in colloids is about 70% and the dominant pore sizes of all samples after drying are below 10 nm). Therefore, the combination of strong surface bonding with buffering effect and spatial confinement allows for regulated freezing behaviors with significantly reduced volume expansion of water in closely packed mineral colloids (Figure 1).

**Figure 1:**
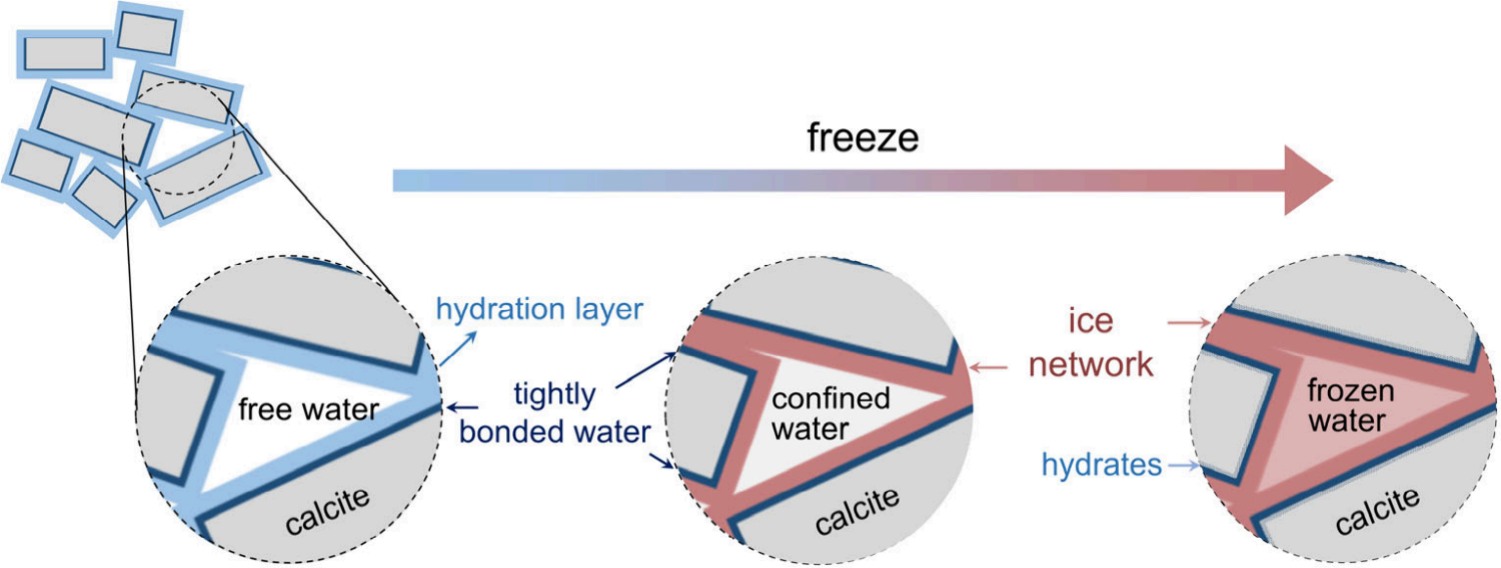
Schematic illustration of the ice-regulating effects achieved using mineral colloids.

Here we take calcite, one of the most abundant minerals on Earth, as an example to verify the above-proposed mechanism. Colloidal calcite was produced by simply mixing the CaCl_2_ and Na_2_CO_3_ solutions, followed by repetitive washing and centrifugation to remove salt in the water phase and to closely pack the colloidal particles (denoted as hydrated calcite colloids, HCC). Characterizations showed that the produced colloidal system possesses the calcite phase (Figure S1a), a particle size range from a few hundred nanometers to approximately 2 μm (Figure S1b), high viscosity and solid-like storage/loss modulus that are typical for colloidal materials (Figure S1c,d), and a total water content of 42.7 wt % (Figure S1e). Raman spectroscopy analysis confirmed the presence of multiple hydration species (Figure 2a). Following previous research,^36^ the water signal at high wavenumbers was divided into five peaks at 3014, 3226, 3432, 3572, and 3636 cm^–1^, corresponding to DAA–OH, DDAA–OH, DA–OH, DDA–OH, and free H_2_O, respectively (D and A indicate donor and acceptor of the hydrogen bond). Notably, the percentage of tetra-coordinated water (DDAA–OH) (also referred to as “ice-like” water) reached 51.1%, which was significantly higher than the DI water (37.4%) (Figure 2b and Figure S2), indicating that calcite particles considerably enhance the content of more ordered “ice-like” water in the colloidal system. The contact angle test in Figure S3 further verifies the enhanced formation of tetra-coordinated water within HCC is not attributed to the hydrophobic surfaces proposed previously^37^ but associated with the hydration ability of calcite itself.

**Figure 2:**
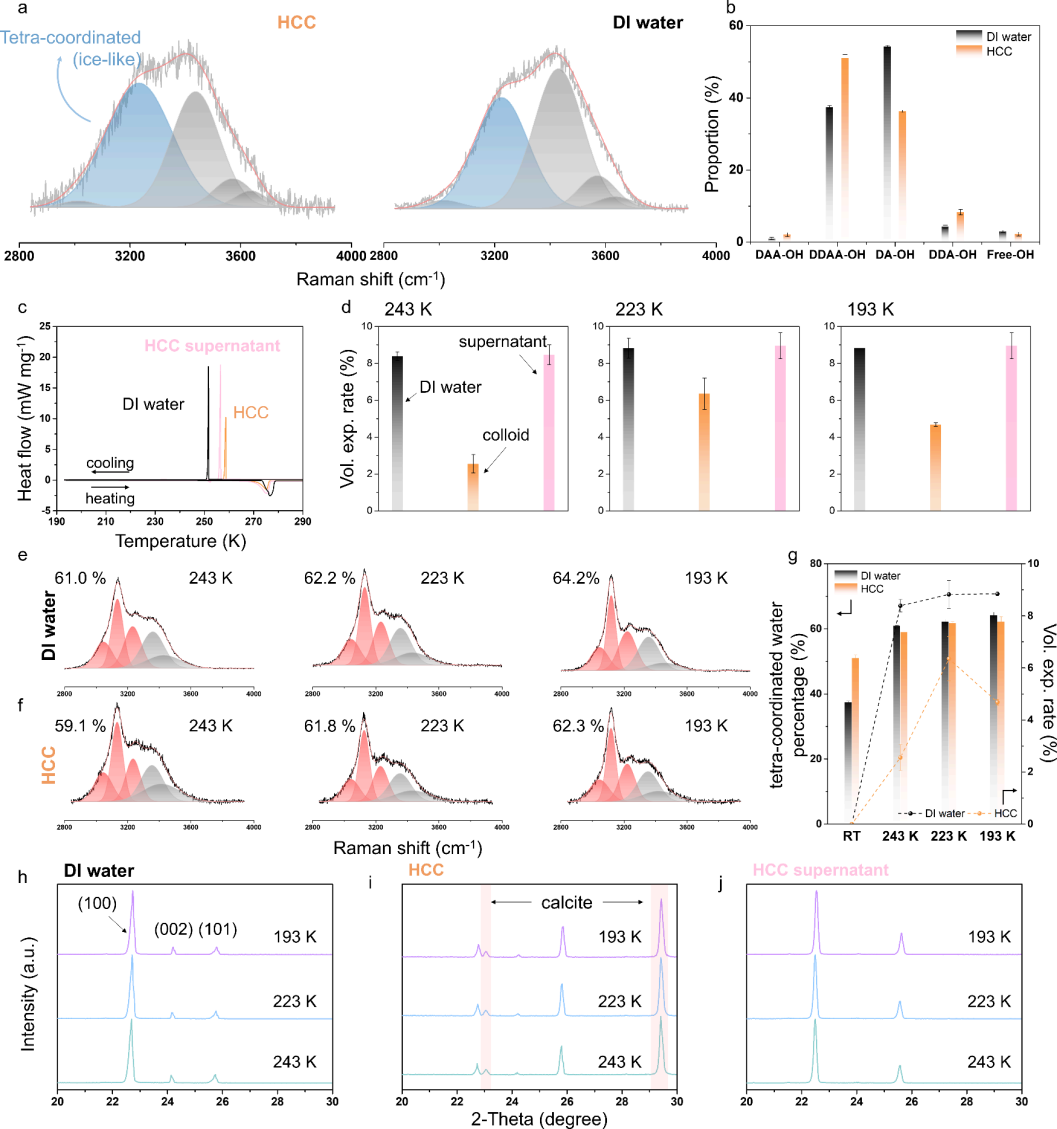
Investigation of hydration characteristics and freezing behaviors of hydrated calcite colloid (HCC) and deionized water (DI water). a) Raman spectra with fitted curves. The blue peak is associated with the vibration of tetra-coordinated (“ice-like”) water molecules. b) The proportion of different coordinated water was calculated based on the three random tests (Figure S2). c) Low-temperature differential scanning calorimetry (DSC) of HCC, the supernatant of HCC, and DI water, cooling from room temperature to 193 K with the rate of 2 K min^–1^. d) Normalized water volume expansion rate upon freezing at 243, 223, and 193 K for DI water, HCC, and the supernatant of HCC. The error bars are standard errors of average volumetric change rates. e,f) Low-temperature Raman spectra of DI water and HCC from 2800 to 4000 cm^–1^. The percentages of the fitted tetra-or-more-coordinated water molecules (red peaks) which were calculated according to three random tests (Figures S4 and S5) were labeled in (e,f) and plotted with the corresponding water freezing expansion rates in (g).^38^ h–j) Low-temperature of X-ray diffraction patterns of DI water, HCC, and HCC supernatant at 243, 223, and 193 K.

Moreover, the differential scanning calorimetry (DSC) measurements found that water in HCC froze at a higher temperature (258.7 K) than the DI water (251.7 K), demonstrating that the presence of calcite particles facilitates the freezing of water (Figure 2c). Interestingly, after frozen at low temperatures, water in HCC displayed much lower volume expansion rates than DI water, with an expansion rate (calibrated according to the water volume ratios) recorded to be only 2.6% at 243 K, compared to 8.4% for DI water at the same temperature (Figure 2d). The volume expansion rate increased at lower temperatures for water in HCC but still significantly lower than DI water: 6.4% at 223 K and 4.7% at 193 K, compared to 8.8% for pure water at both temperatures. To confirm the above-observed reduced volume expansion, another displacement method that employed ethanal as the soaking media was used, showing similar measurement results (Figure S6).

In-situ Raman techniques were used to study the bonding states and lattice structures during the cooling process (Figure 2e,f). Upon cooling, HCC and DI water both exhibited an increase in the content of tetra-coordinated water molecules.^39^ It is well documented that the volume expansion of water upon freezing into ice is due to the repulsion caused by the formation of additional hydrogen bonds or tetra-coordinated water. First, water in HCC had fewer hydrogen bonds forming upon freezing than DI water, indicating that its relatively lower degree of structural rearrangement or repulsion occurs upon freezing (Figure 2g). Moreover, from the normalized amount of tetra-or-more-coordinated water molecules and the calculated contribution coefficients of the formation of hydration layer (eq S4, Table S1), it can be seen that the hydration water of tetra-coordinated hydrogen bonds is critical for enabling the antiexpansion effect. The lattice structures of ice at different temperatures were recorded in X-ray diffraction patterns (Figure 2h–j and Figure S7). The growth of ice in HCC was distinct from that of DI water or its supernatant, showing a noticeable orientation preference: The (101)_*Ih*_ peaks were significantly higher than the (100)_*Ih*_ ones, contrary to the behavior observed on ice and the supernatant (Figure 2h–j). This pattern is similar to those seen in antifreezing polymers, which also enhance the prominence of the (101)_*Ih*_ peak,^40^ indicating the strong ice-regulating effect of HCC.

For comparison, saline calcite colloids (SCC) were synthesized using the same method (e.g., centrifuged at 5000 rpm after reaction to precipitate the colloidal particles) but without additional washing with DI water. SCC exhibited the same phase of calcite as HCC (Figure S8a), but a lower viscosity and a lower content of tetra-coordinated water (Figure S8b–d) (the exact concentration of tetra-coordinated water was calculated according to three random tests in Figure S9), revealing that the existence of the sodium and chloride ions in the liquid phase weakened the hydrogen bonding between calcite and its surface hydration and thus its antiexpansion capability. Expectedly, relatively higher water expansion rates upon freezing were achieved by SCC (the lowest being 5.6%, observed at 193 K) (Figure S8e). Similarly, loosely packed SCC and HCC (LHCC and LSCC), which were obtained by using a lower centrifuge speed of 2000 rpm, possessed lowered viscosities, decreased contents of tetra-coordinated water, and hence worsened capability to suppress the freezing expansion of water (Figure S10). Besides, based on the tetra-coordinated water’s concentration and expansion rate of DI water, LHCC, and HCC, a linear relationship was revealed (Figure S11): as the amount of tetra-coordinated water rises, the expansion volume rate decreases linearly.

From the above characterizations, the tightly bonded surface hydration and closely packed morphology are counted for the underlying freezing-regulation mechanism of mineral colloids. Specifically, the innermost water layers tightly bonded to the mineral surface possess a high content of tetra-coordinated configurations and a low density (the deep blue layer in Figure 2a).^41^ They can be viewed as nonfreezable and thus do not make an appreciable contribution to volume expansion upon freezing. Meanwhile, featuring a more ordered “ice-like” structure, these innermost interfacial hydrations offer a ubiquitous large working surface throughout the colloidal material to facilitate the energetically favored heterogeneous nucleation (as evidenced by the fact that HCC can induce a higher freezing point than DI water or its supernatant, Figure 2c). Furthermore, the outer moderately bounded hydration layers possess more ordered structures less dense than free water and can readily freeze upon cooling. These factors, along with the close contact among the surface hydration layers, facilitate the rapid formation of a continuous ice network when the temperature drops, leaving some free water trapped in the interstitial spaces among the mineral particles (Figure 1). Under this condition, internal growth of ice received a compression from outer concurrent ice structure, which is revealed by the positive relationship between locations and angle’s increasement in Figure S12. These interstitial water molecules are not only confined but also possibly compressed by the ice network, due to the volume expansion caused by the freezing event of the hydration water, hindering their transformation into crystalline ice (*I*_h_).

Note that, to enable the formation of the continuous ice network, the colloidal particles need to be closely packed to allow their surface hydration layers to come in close contact. This is consistent with the above observation that loosely packed HCC and SCC did not offer the same capability to suppress water expansion upon freezing. To further verify this mechanism, in situ XRD measurements were used to monitor the freezing process of HCC and LHCC (Figure 3a,b). The initial appearance of the (101)_*Ih*_ peak before other peaks, resulting from a higher content of tightly bonded hydration water (as for HCC), was recognized as an indicator for the interfacial crystallization of water. The (101)_*Ih*_ peak emerged first (1 K ahead of the (100)_*Ih*_ and (002)_*Ih*_ features) and stayed as the strongest peak for HCC (as shown in Figures 2i and 3a,b), whereas the (101)_*Ih*_ and (100)_*Ih*_ peaks appear simultaneously for LHCC. This sharp contrast indicates that a close-packed morphology is essential for directing the initial ice crystallization exclusively at the mineral surface.

**Figure 3:**
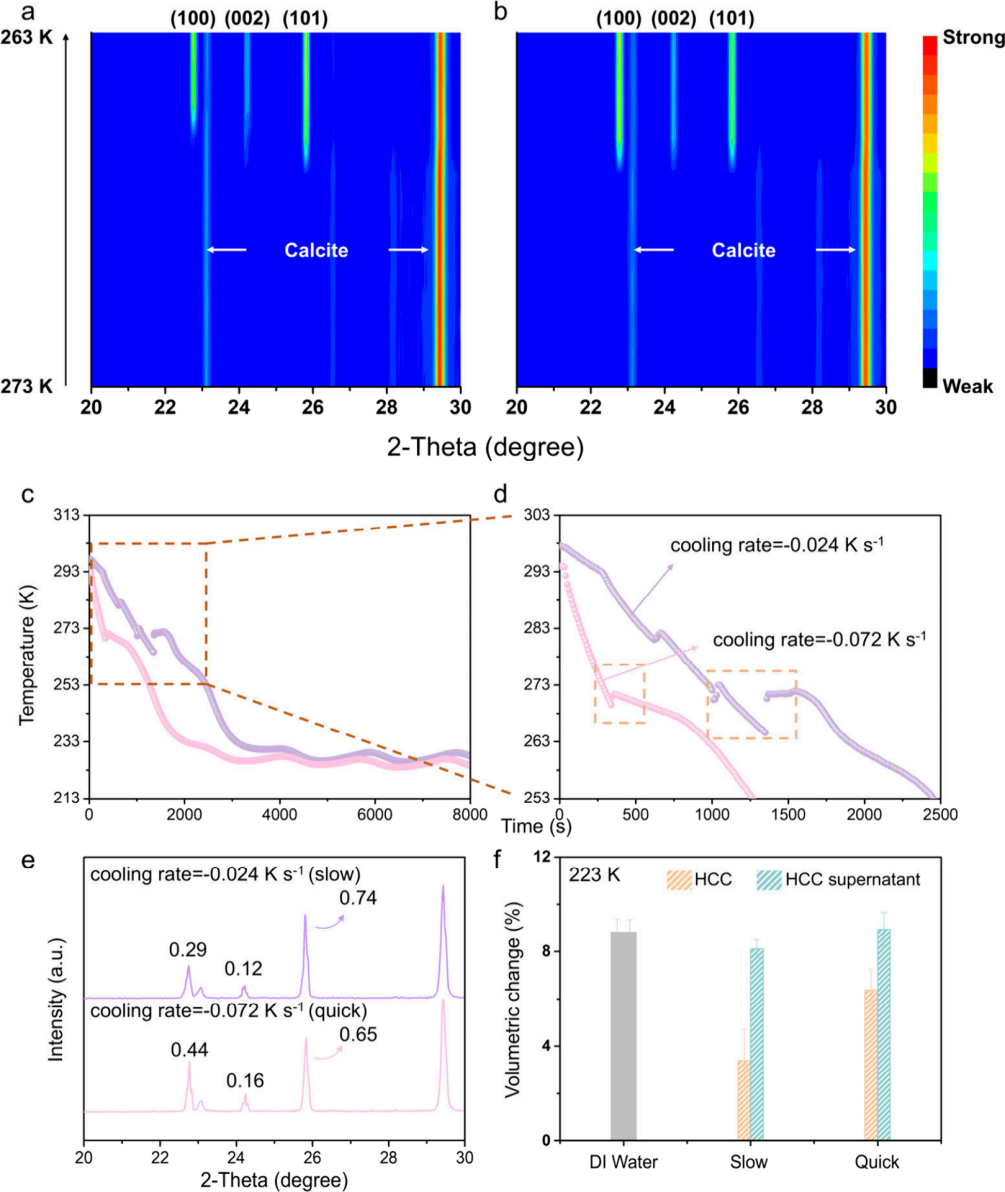
Freezing behavior study. a,b) Initial freezing behavior of HCC (a) and LHCC (b), monitored using in situ X-ray diffraction measurements. The cooling rate and scanning rate are −1 K min^–1^ and 10° min^–1^, respectively. The color bar on the right is the reference to the intensity of the diffraction signal. c) Temperature-cooling time curve with different cooling rate. d) Enlarged version of temperature-cooling time from 0 to 2500 s. e) X-ray diffraction patterns and volumetric change of HCC freezing at 223 K with different cooling rates. The numbers in the X-ray diffraction patterns are the relative intensity (normalized by the intensity of calcite (104) facet) of three main peaks of ice. f) Volumetric change with different cooling rate. The left bars in the same group (slow or quick) represent the HCC, while the right ones are the corresponding supernatant.

According to the classic nucleation theory, compared to the homogeneous nucleation, the heterogeneous nucleation lowers the energy barrier for forming a critical nucleus for crystal growth by utilizing foreign surfaces, leading to the requirement of a lower undercooling degree, which is desirable to study the contribution of the hydration layer or the surface of mineral colloid. Inspired by this idea, a slow, stepwise cooling approach was employed to optimize the freezing behavior of HCC at the target temperature of 223 K. The cooling rates were recorded using a temperature sensor with an interval of 10 s: 0.072 K s^–1^ for fast cooling and 0.024 K s^–1^ for slow cooling (Figure 3c,d). HCC displayed more sluggish freezing kinetics upon slow cooling. Observations further showed that the (101)_*Ih*_ peak—an indicator of interfacial heterogeneous nucleation—exhibited a stronger normalized intensity in the slowly cooled sample compared to the rapidly cooled sample (Figure 3e). Furthermore, the water freezing expansion rate decreased from 6.4% to 3.3%. Based on the classical nucleation theory, heterogeneous nucleation is more effective at initiating crystallization, or freezing, at lower degrees of supercooling, whereas homogeneous nucleation requires higher degrees of supercooling. Consequently, in experiments utilizing a slow, stepwise cooling approach, which imposes a lower degree of supercooling at each step, heterogeneous nucleation achieves a higher nucleation priority compared to homogeneous nucleation in the initial direct cooling experiments. With this difference, the reduction of expansion provides additional evidence that improved heterogeneous nucleation is crucial to the antiexpansion capability (Figure 3f). This supports the previously discussed mechanism.

To obtain more insights into the hydration-mineral interaction, the composition, surface valence states, and lattice arrangement of freeze-dried HCC were compared with those of heat-dried HCC (343 K for 120 h). Previous research has demonstrated that water molecules can infiltrate the mineral lattice and form mineral hydrates under pressure (i.e., to undergo the chemical reaction of hydration).^31^ As a readily hydrated mineral,^42^ calcite is likely to react with water under pressure, producing various hydration species at the water-calcite interface. The thermogravimetric measurement showed a higher content of tightly bound water, obtained from the weight loss between 423 to 673 K, for freeze-dried HCC (Figure 4a), indicating the formation of hydrated minerals from freezing.

**Figure 4:**
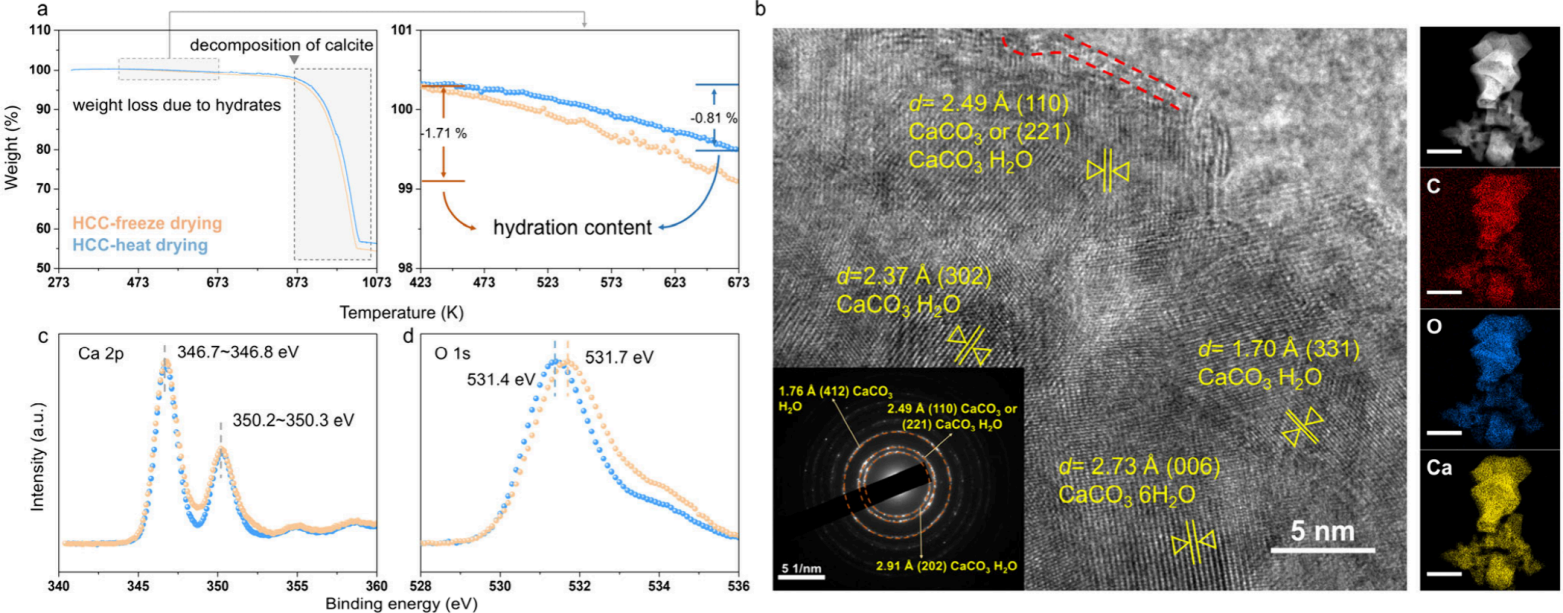
Characterization of freeze-dried HCC and heat-dried HCC. a) TG curves and enlarged version ranging from 423 and 673 K. b) High-resolution transmission electron microscope images with selective area electron diffraction pattern and elemental distribution spectra mapping (unlabeled scale bars indicate 250 nm) of freeze-dried HCC. c,d) X-ray photoelectron spectra of Ca 2p and O 1s.

Exotic surface hydrate species of the freeze-dried HCC were revealed by X-ray photoelectron spectroscopy (XPS) and high-resolution transmission electron microscopy (HRTEM) (Figure 4b–d and S13). Compared with the XPS spectra of heat-dried HCC, freeze-dried HCC exhibited only minor fluctuations (up to 0.1 ∼0.2 eV) for the C and Ca peaks, but distinct O 1s features, suggesting the distinct chemical states of its carbonate ions and pointing to the presence of ample bound water within the calcite lattice of the freeze-dried HCC.^43,44^ Furthermore, HRTEM of freeze-dried HCC spotted lattice features characteristic of hydrated calcite (Figure 4b), which is consistent with the monohydrate calcite phase detected from the selective area electron diffraction (SAED) patterns (Figure 4b). All of the above observations suggest that a variety of hydrated calcium carbonate products were produced during the freezing process of the closely packed HCC.

To assess the universality of the ice-regulation mechanism observed with minerals, another most common mineral, calcium phosphate, was synthesized into closely packed saline colloids (denoted as SCPC) and hydrated colloids (HCPC, Figure S14), whose viscosities exceed 40000 and 60000 Pa s, respectively. In comparison to HCC, both SCPC and HCPC exhibited a lower content of ice-like hydration (21.5% and 31.4%, respectively) (Figure S14g,h) as well as higher expansion rates during water freezing. For instance, the minimum expansion rate of 3.2% was recorded for HCPC at 243 K, which is 1.3 times greater than that of HCC at the same temperature (Figure S14d). These differences can be attributed to the weaker hydration capability of calcium phosphate compared to calcite,^45,46^ which further emphasizes the importance of hydration capacity instead of the structural constraint for reducing expansion to a smaller value.

Besides, the particle sizes of all the calcite and phosphate mineral colloids studied in this work range from submicron to 100 μm, as measured using the dynamic light scattering method (Figure S15). For calcite systems, a higher centrifugation rate is able to reduce the particle size, while the washed colloids possess a larger particle size, suggesting the existence of sodium or chlorine ions would increase the surface charge. Interestingly, for tricalcium phosphate, the washed colloids display a smaller particle size, indicating a different influence of the dissolved salt ions on the surface properties of the phosphate colloids. The surface area and the pore size distribution were characterized using nitrogen adsorption/desorption (Figure S16). The dominant pore sizes of all samples are below 10 nm. For calcite colloids, the smaller particle size (as revealed by dynamic light scattering, DLS) does render higher surface areas. Interestingly, the tricalcium phosphate colloids exhibit a larger surface area in spite of their larger particle sizes (as revealed by the above DLS results), suggesting the porous nature of these particles.

In summary, this study reports the remarkable ice-regulating properties of minerals and reveals the important role of surface hydration in the antiexpansion mechanism. We discovered that purely inorganic mineral colloids can effectively suppress the volume expansion rate of water upon freezing at low temperatures (e.g., 8.4% lowered to 2.6% at 243 K) due to a new mechanism: The interfacial water layers on the mineral surfaces feature a more ordered “ice-like” structure and form a continuous network in a colloidal system with closely packed mineral particles. Upon cooling, they concurrently freeze into an ice framework with the aid of heterogeneous nucleation, accompanied by less volume expansion, while confining the interstitial free water. Therefore, minerals, the most ubiquitous, abundant, diverse, and “dirt-cheap” materials on Earth, are now added to the family of ice-regulating materials, bringing fresh insights into the various water-related fields, such as mineralogy, geology, astronomy, biomineralization, and cryopreservation.

## Supplementary Material



## Data Availability

Data will be made available on request.

## Notes

Information regarding Materials and Methods; Structural information on hydrated calcite colloid (HCC); Raman spectra of DI water and HCC with fitted curves; Contact angle measured on the tablets made by freeze-dried HCC and SCC powders; Raman spectra of water with fitted curves at 243, 223, and 193 K; Raman spectra of HCC with fitted curves at 243, 223, and 193 K; Normalized volumetric expansion rate according to Archimedes’ principle with ethanol; In-situ X-ray diffraction pattern of HCC recorded at different temperatures; Structural information on saline calcite colloid (SCC); Raman spectra of SCC with fitted curves; Structure information and the freezing behaviors of loosely packed HCC and SCC (LHCC and LSCC); Linear fitting conducted on the data of DI water, LHCC, and HCC; Investigation of XRD peaks migration of HCC at 243 K; High-resolution C 1s spectra (XPS) of freeze-dried HCC (orange) and heat-dried HCC (blue); Structure information on tighter calcium phosphate colloids (SCPC and HCPC) and the freezing behavior; Particle size distribution of colloids; Nitrogen adsorption/desorption curves and pore size distribution of colloids; Fitting and extrapolation of ethanol’s density; Contribution coefficient table of tetra-or-more-coordinated hydration layer to the reduced water; Table containing Packing density, water content, and viscosity of calcite colloids. (PDF)
