# Supplementary material for: Concurrent Formation of Ice Network within Mineral Colloids with Suppressed Volume Expansion
Source: J Phys Chem Lett. 2026 Jan 19;17(4):991–9. doi: 10.1021/acs.jpclett.5c03627 (PMC12862801; doi:10.1021/acs.jpclett.5c03627)
Supplement: Supplementary file 1 [file jz5c03627_si_002.pdf]

Supplementary materials for

Concurrent formation of ice network within mineral colloids with suppressed volume expansion

Hongkun Li,[1,2,3,4] Yunchen Long,[1,2,3] Junda Shen,[1,2] Xinxue Tang,[1,2,3,4] Jiahua Liu,[1,2,3,4] Chong Wang,[1,5] Binbin Zhou,[6] Bo Li,[1,2,3] Jing Zhong,[1,2,3] Xiao Ma,[7] Chunyi Zhi,[3,8,*] Jian Lu,[1,2,3,4,*] Yang Yang Li [1,2,3,4,*]

[1] *Hong Kong Branch of National Precious Metals Material Engineering Research Center, City University of Hong Kong, Hong Kong SAR, China*

[2] *Centre for Advanced Structural Materials, City University of Hong Kong Shenzhen Research Institute, Greater Bay Joint Division, Shenyang National Laboratory for Materials Science, Shenzhen 518057, China*

[3] *Department of Materials Science and Engineering, City University of Hong Kong, Hong Kong SAR, China*

[4] *Department of Mechanical Engineering, City University of Hong Kong, Hong Kong SAR, China*

[5] *Department of Materials Science and Engineering, College of Transportation Engineering, Dalian Maritime University, Dalian, 116026, PR China*

[6] *Shenzhen Institute of Advanced Electronic Materials, Shenzhen Institute of Advanced Technology, Chinese Academy of Sciences, Shenzhen 518055, China*

[7] *Department of History of Science and Scientific Archaeology, University of Science and Technology of China, Hefei, 230026, China*

[8] *Department of Mechanical Engineering, The University of Hong Kong, Hong Kong SAR, China*

* Corresponding authors: cyzhi@hku.hk; jianlu@cityu.edu.hk; yangli@cityu.edu.hk

Materials and Methods

Synthesis and freezing of mineral gel

Colloidal calcite was synthesized by mixing the aqueous solutions of calcium chloride (Sigma, AR, 10043-52-4) and sodium carbonate (Sigma, AR, 497-19-8) and stirring in DI water (the final concentration is 2 M for both calcium chloride and sodium carbonate) for 30 minutes, followed by centrifugal washing (typically at 5000 rpm and 2000 rpm) for 3 times to remove the soluble salts in the water phase. The colloidal packing densities, water content and viscosity were listed in **Table S2**. Similarly, colloidal tri-calcium phosphate was prepared by mixing solutions of calcium chloride (Sigma, AR, 10043-52-4) and tri-potassium phosphate (Sigma, AR, 7778-53-2) and stirring in DI water (the final concentration is 3 M for calcium chloride and 2 M tri-potassium phosphate) for 30 minutes, followed by centrifugal washing by DI water (typically at 5000 rpm) for 3 Times. Their micro morphology is characterized by the scanning electron microscope. For fast freezing treatment, the sample (1.4 to 4 ml) was directly incubated at the target temperature for 24 hours, whereas slow freezing was carried out by gradually cooling the sample at different temperatures (278, 266, 255, and 243 K, each maintained for 5 min) before leaving the sample at 223 K for 24 hrs. The cooling rate is also recorded by temperature sensor with an interval of 10 s (**Fig. 3c-d**). The volumetric ratio of water (R_w) in the colloidal system is calculated from its weight loss at 200 ^{o}C for 2 hrs and the densities of calcite (2.71 g cm^{-3}), tricalcium phosphate (3.14 g cm^{-3}), water (1.00 g cm^{-3}), NaCl (2.16 g cm^{-3}) and KCl (1.98 g cm^{-3}).

Characterizations

Scanning electron microscopy (SEM) study was performed on a Thermo Scientific Quattro ESEM system. All the samples were dried at 353 K for 24 hrs first and sputtered with Au for 30 sec in advance. Low-temperature X-ray diffraction (XRD) patterns were collected on a Bruker D8 Discover diffractometer with Cu radiation under 40 kV/40 mA. To monitor the structural evolution, XRD patterns were collected on during the first 30 minutes after reaching the target temperatures of 243, 223, and 193 K with an interval of 3 min. To characterize the initial freezing behavior of the sample, the cooling rate was reduced to -1 K per minute, while the XRD scanning range was set to be 20-30° with a scanning rate of 10° min^{-1}, i.e., the freezing process was measured once a minute. Raman spectra are carried out at both room and low temperatures with the excitation laser wavelength of 532 nm and laser power of 10 mW. The viscosity and modulus (storage and loss) of the sample (1mm thickness) were measured using the Kinexus rheometer. The viscosity was tested through shear rate mode from 0.1 to 10 s^{-1} at 298 K. The modulus was

characterized using 0.1% strain with the frequency ranging 0.1-100 hz. Differential scanning calorimetry was carried out from room temperature to 193 K with a cooling rate of 2 K min^{-1}. The adsorption/desorption curves, specific surface area, and the distribution of pore size were analyzed using automatic surface area and pore size analyzer (ASAP2460). All the samples for the surface area measurement were freeze-dried first and then degassed for at least 8 hrs. The lattice arrangement and elemental distribution were obtained using the transmission scanning microscope of JEM-F200 equipped with JED-2300. The Mettler Toledo TGA/DSC 3+ instrument and PerkinElmer STA 6000 Simultaneous Thermal Analyzer was used to characterize the total amount of water in calcite colloid, and the hydration of calcite with different drying methods with a heating rate of 10 K min^{-1}. The valence state changes were recorded by Thermo Fisher ESCALAB XI^{+} X-ray Photoelectron Spectrometer. The particle size distribution of calcite or phosphate-based colloids was investigated by dynamic light scattering method (Malvern Mastersizer 2000). Before the measurement, a ten-minutes ultrasonication was applied to disperse the powder. The surface area and pore distribution of colloids were studied using Micromeritics ASAP 2460, and the degas process was conducted at 423 K for 8 hours. The contact angle tests were carried out on DataPhysics Contact Angle Tester. Before tests, the freeze-dried sample powders were compressed by 300 MPa for 3 minutes.

Volumetric measurement at low temperature

Method-1

To obtain the mineral colloidal volume before and after freezing, a volume deduction method was developed. Through measuring the volume of supernatant after the centrifugation of mineral-dispersed solution whose volume is set to 7 ml initially, the original volume of colloids (V_{O}) can be calculated as follows,

$$V_{\mathrm{O}} = V_{\mathrm{D}} - V_{\mathrm{S}} \qquad \textbf{eq. s1}$$

where V_{D} is the volume of mineral-dispersed solution and V_{S} is the volume of supernatant after centrifugation. To obtain the volume after freezing, DI water with recorded volume was added to the frozen samples until a target volume within 20 s, then the freezing volume (V_{F}) is

$$V_{\mathrm{F}} = V_{\mathrm{T}} * R_{\mathrm{S}} - V_{\mathrm{W}} \qquad \textbf{eq. s2}$$

where V_{T} is the final total volume after adding water, R_{S} is the shrinkage rate of tube (about 0.991) and V_{W} is the volume of added water. Then the volumetric change percentage (P_{C}) is calculated. The averaged volumetric change percentage $P_{\mathrm{C,A}}$ is normalized by the volumetric ratio of water, R_{W},

$$P_{\mathrm{C,A}} = \frac{P_{\mathrm{C}}}{R_{\mathrm{W}}} = \frac{V_{\mathrm{F}}-V_{\mathrm{O}}}{V_{\mathrm{O}}*R_{\mathrm{W}}} * 100\ \% \qquad \textbf{eq. s3}$$

The contribution coefficient of the hydration layer to the reduced volume expansion was calculated:

$$C = \frac{R_{\mathrm{T,HCC}}-R_{\mathrm{0,HCC}}}{R_{\mathrm{T,water}}-R_{\mathrm{0,water}}} * \frac{P_{\mathrm{T,E,water}}}{P_{\mathrm{C,A}}} * 100\ \% \qquad \textbf{eq. s4}$$

in which $R_{\mathrm{T,HCC}}$, $R_{\mathrm{0,HCC}}$, $R_{\mathrm{T,water}}$, and $R_{\mathrm{0,water}}$ stand for the ratio of tetra- or more coordinated water molecules in HCC and pure water at the testing temperature and the room temperature, respectively. The $P_{\mathrm{T,E,water}}$ is the expansion percentage recorded at the same temperature in pure water.

Method-2

The volumetric changes of all mineral colloids, supernatant, and DI water were also measured using the displacement method that employed ethanol as the soaking media. The density of Ethanol used in the calculation were obtained through fitting and extrapolation to reported data[1,2] (**Fig. S17**). The calculation equations are listed as below,

$$P = \frac{\left(\frac{m_{air}-m_{ethanol}}{d_{ethanol,T}}-V_O\right)}{V_O}/R_w * 100\ \% \qquad \textbf{eq. s5}$$

in which *P* stands for the volumetric change of sample, including DI water, mineral colloids, and corresponding supernatant at the testing temperature. m_{air} and $m_{ethanol}$ represent the weight measured in the air or ethanol. The used $d_{ethanol,T}$ is calculated according to fitting result in **Fig. S17.** V_{O} is the original volume of colloids and R_{W} is the volumetric ratio of water.

Raman deconvolution method

For the spectra obtained at room temperature, deconvolution is conducted using Gaussian methods between 2750 and 3950 cm^{-1}, with five peaks located at 3014, 3226, 3432, 3572, and 3636 cm^{-1}.[3] The default adjacent-averaging smoothing method was used for the baseline anchor point. The value of the adj R^2 of the fitting results is about 0.921-0.999, which is affected by the signal-to-noise ratio.

For the spectra obtained at sub-zero temperatures, deconvolution is conducted using Gaussian methods within the range of 2800 to 3980 cm^{-1}, with five peaks located at 3050, 3128, 3236, 3360, and 3430 cm^{-1}.[4] The default adjacent-averaging smoothing method was used for the baseline anchor point. The value of adj R^2 of the fitting results is about 0.935-0.998, which is affected by the signal-to-noise ratio.

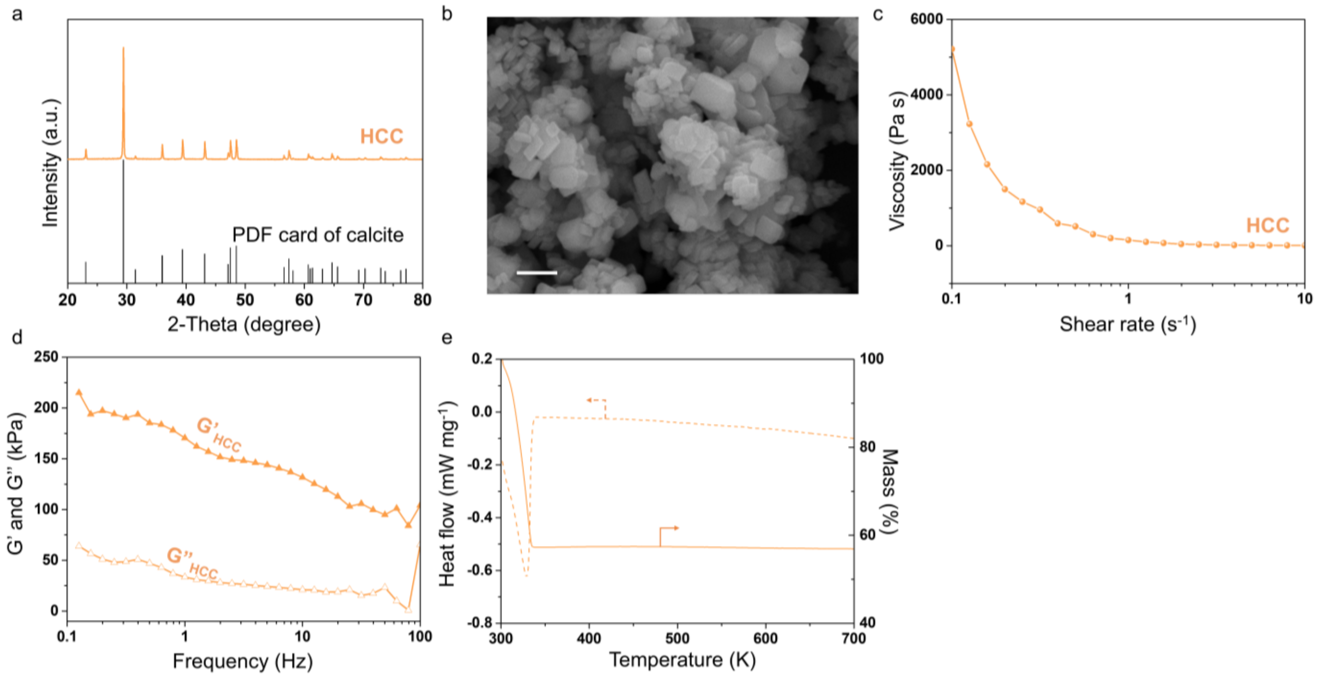

Figure S1. Structural information of hydrated calcite colloid (HCC). a) X-ray diffraction patterns of HCC and standard PDF card of calcite. b) Scanning electron microscopy (SEM) image. The scalebar used in SEM image is 2 µm. c)Viscosity. d) Storage (G') and loss (G'') modulus. e) Thermogravimetric analysis (TGA) and differential scanning calorimetry (DSC) curves measured with a heating rate of 10 K min^{-1} and a nitrogen flow.

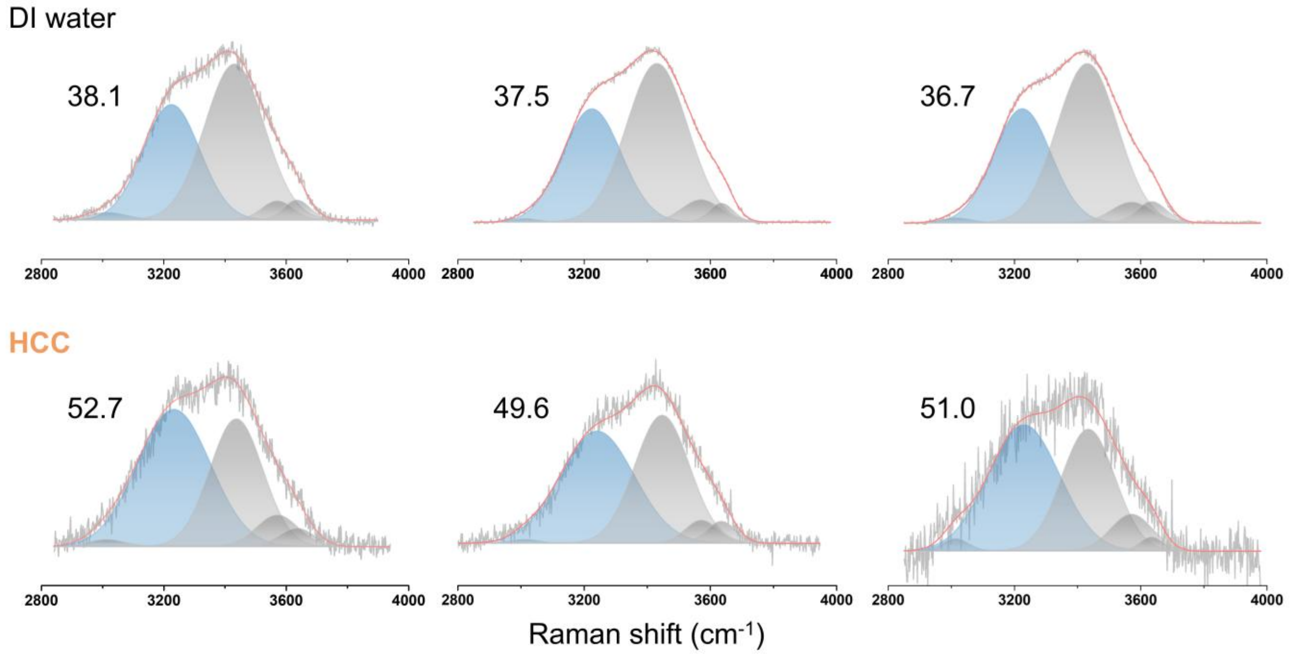

Figure S2. Raman spectra of DI water and HCC with fitted curves. The first and second rows of graphs belong to DI water and HCC, respectively. All the spectra are activated by a laser of 532 nm and taken at ambient temperature. The inserted number is the ratio of tetra-coordinated water whose sub peak is labelled by blue.

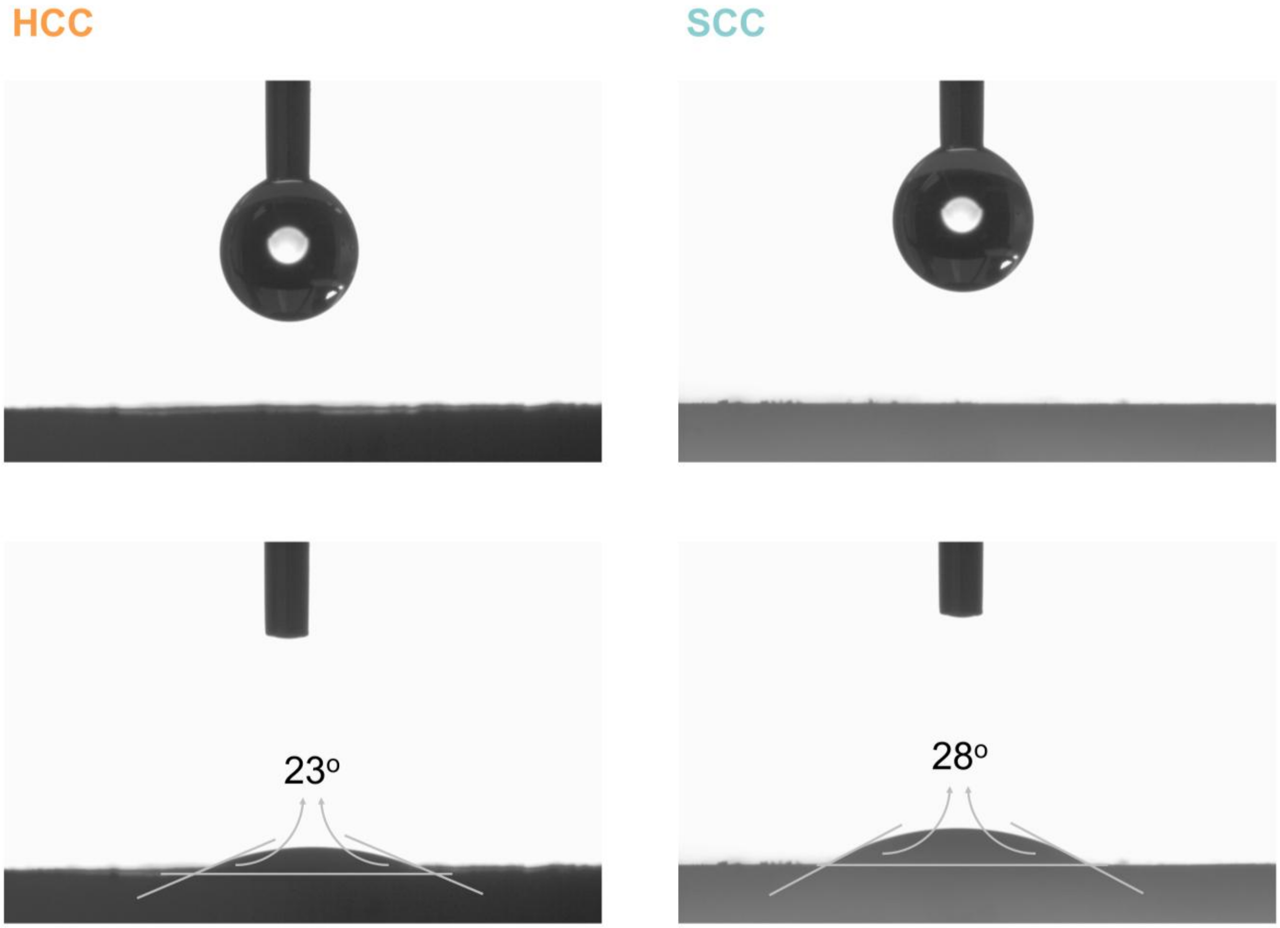

Figure S3. Contact angle measured on the tablets made by freeze-dried HCC and SCC powders. The second line figures were taken at 3 seconds after the water came into contact with the sample surface and then the droplets were immersed into tablets.

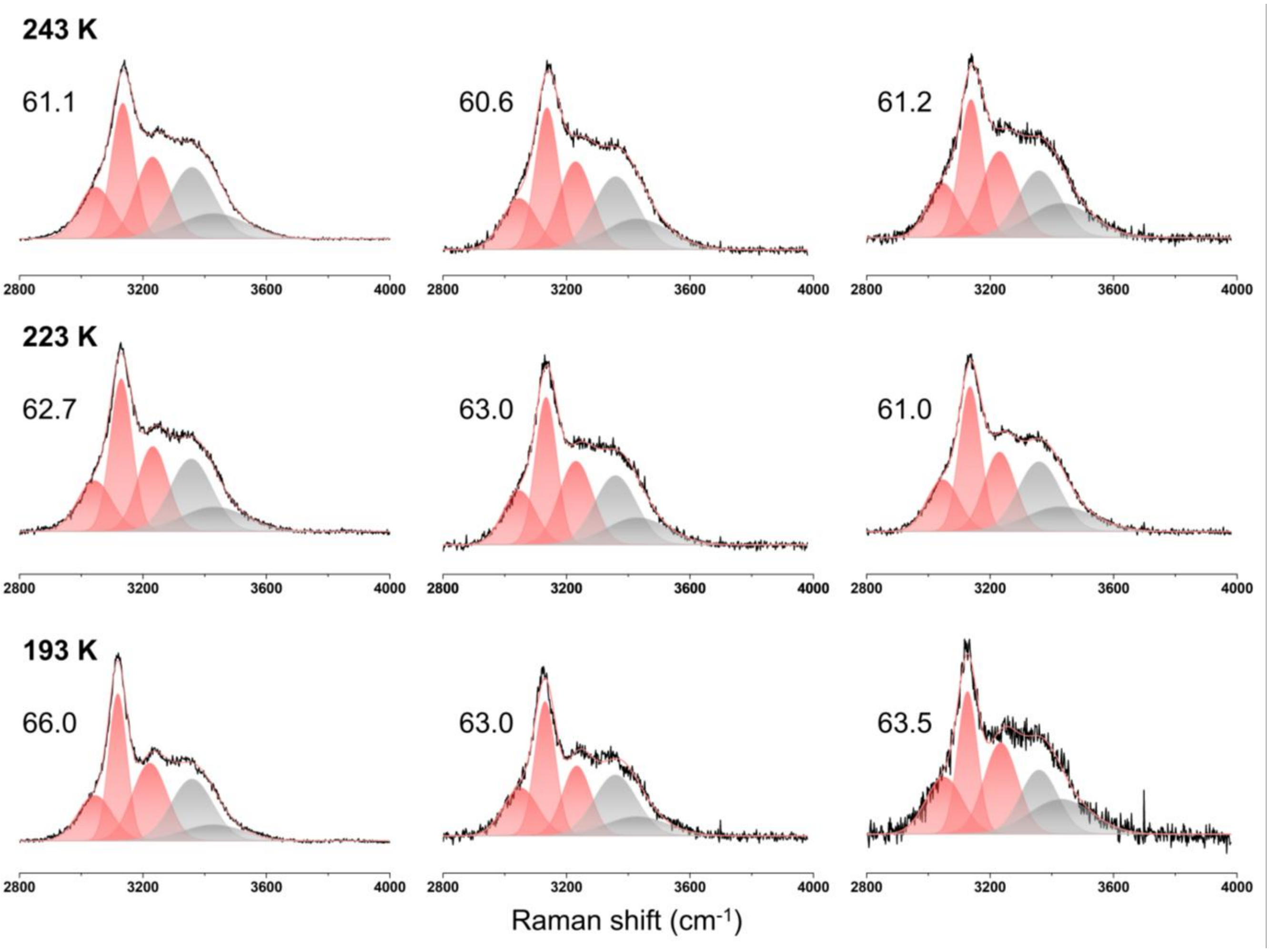

Figure S4. Raman spectra of water with fitted curves at 243, 223, and 193 K. The acquisition temperature of each row of spectra is listed in the upper left corner. All the spectra are activated by a laser of 532 nm. The marked number is the ratio of tetra-coordinated water which is consist of the three red sub peaks.

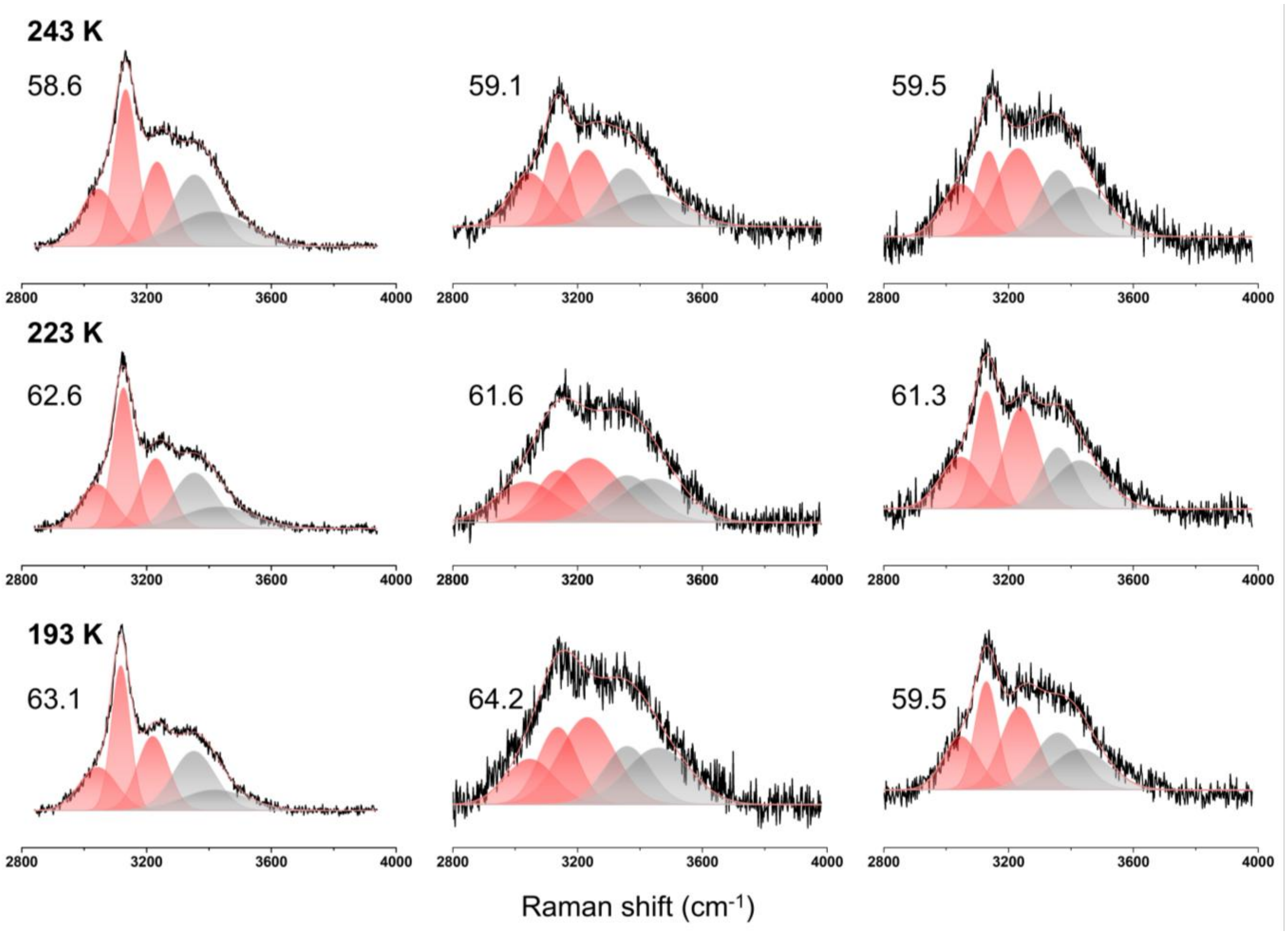

Figure S5. Raman spectra of HCC with fitted curves at 243, 223, and 193 K. The acquisition temperature of each row of spectra is listed in the upper left corner. All the spectra are activated by a laser of 532 nm. The marked number is the ratio of tetra-coordinated water which is consist of the three red sub peaks.

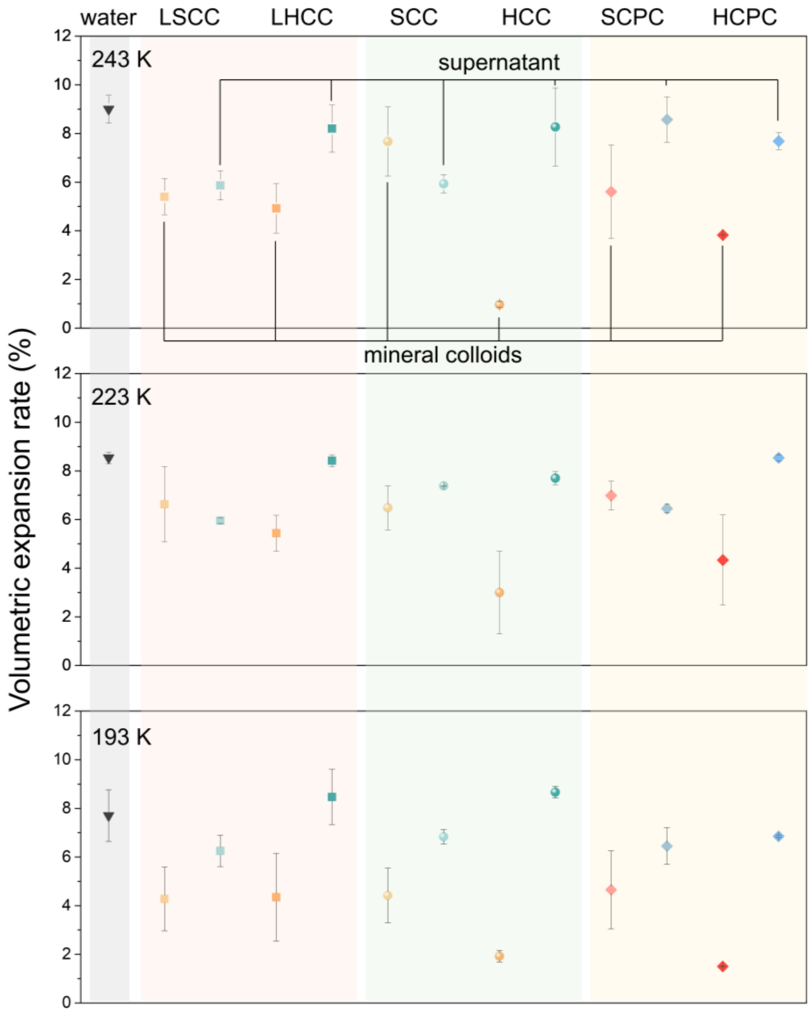

Figure S6. Normalized volumetric expansion rate of mineral colloids and corresponding supernatant recorded according to Archimedes' principle with ethanol at 243 K, 223 K, and 193 K. The expansion rate is All the error bars are the standard errors of average volumetric change rates.

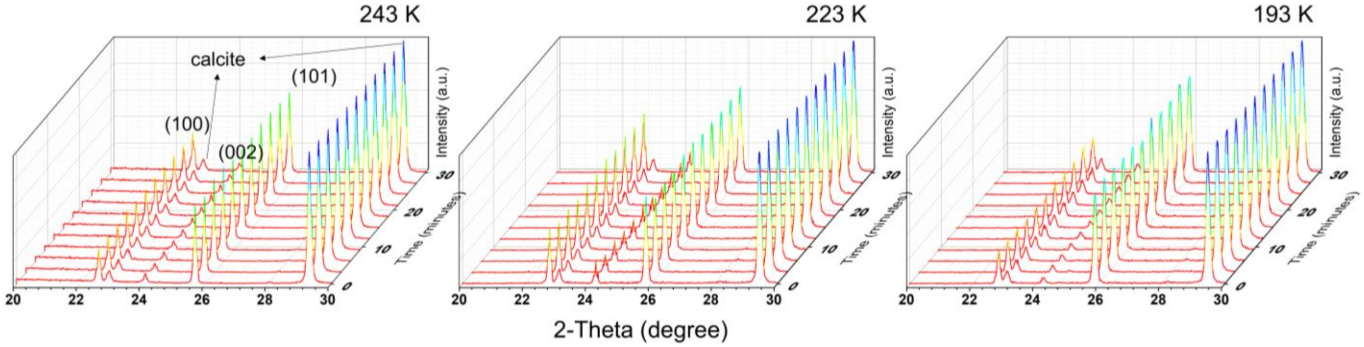

Figure S7. In-situ X-ray diffraction pattern of HCC recorded at different temperatures from 0 to 30 minutes, the five peaks from low to high 2-Theta value stand for the $(100)_{Ih}$, (012) (calcite), $(002)_{Ih}$, $(101)_{Ih}$, and the dominating plane of calcite (104) respectively.

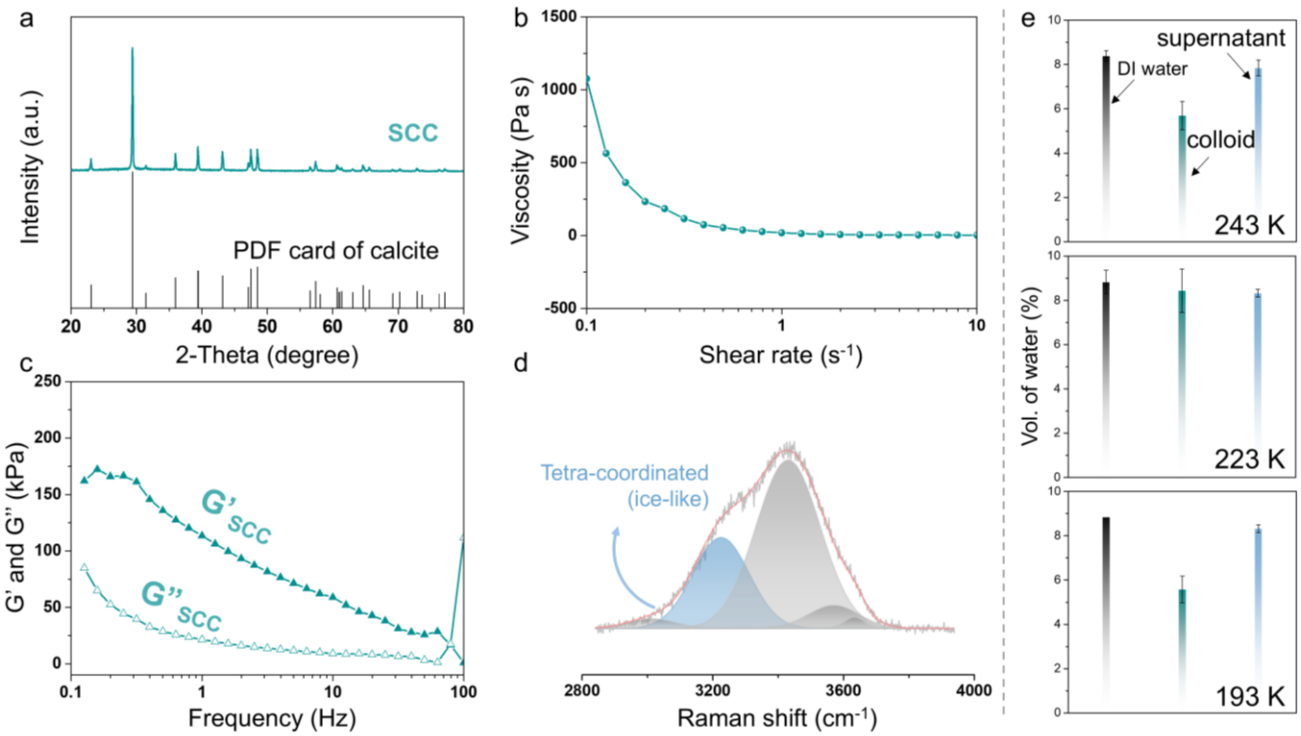

Figure S8. Structural information of saline calcite colloid (SCC). a) X-ray diffraction patterns of SCC and standard PDF card of calcite. b) Viscosity of HCC. c) Storage (G') and loss (G'') modulus of HCC. d) Raman spectra and fitted curves, and the blue sub peak stands for the tetra-coordinated water molecules. e) Expansion rate of SCC at different temperatures (243 K, 223 K, and 193 K).

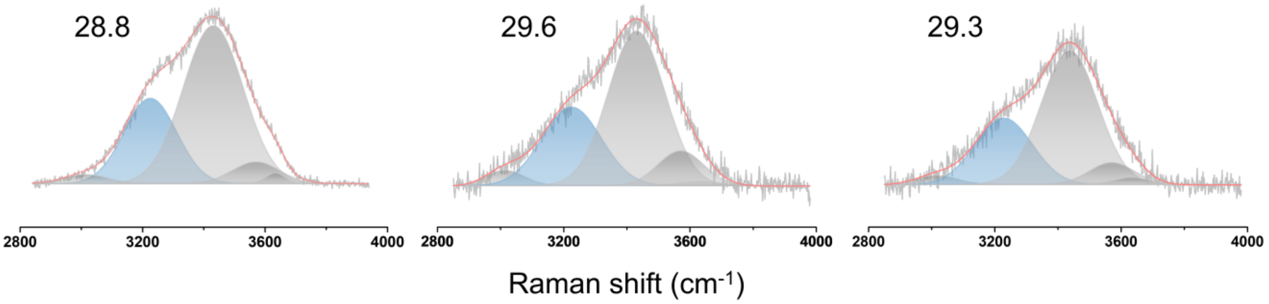

Figure S9. Raman spectra of SCC with fitted curves. All the spectra are activated by a laser of 532 nm and taken at ambient temperature. The inset number is the ratio of tetra-coordinated water whose sub peak is marked by blue.

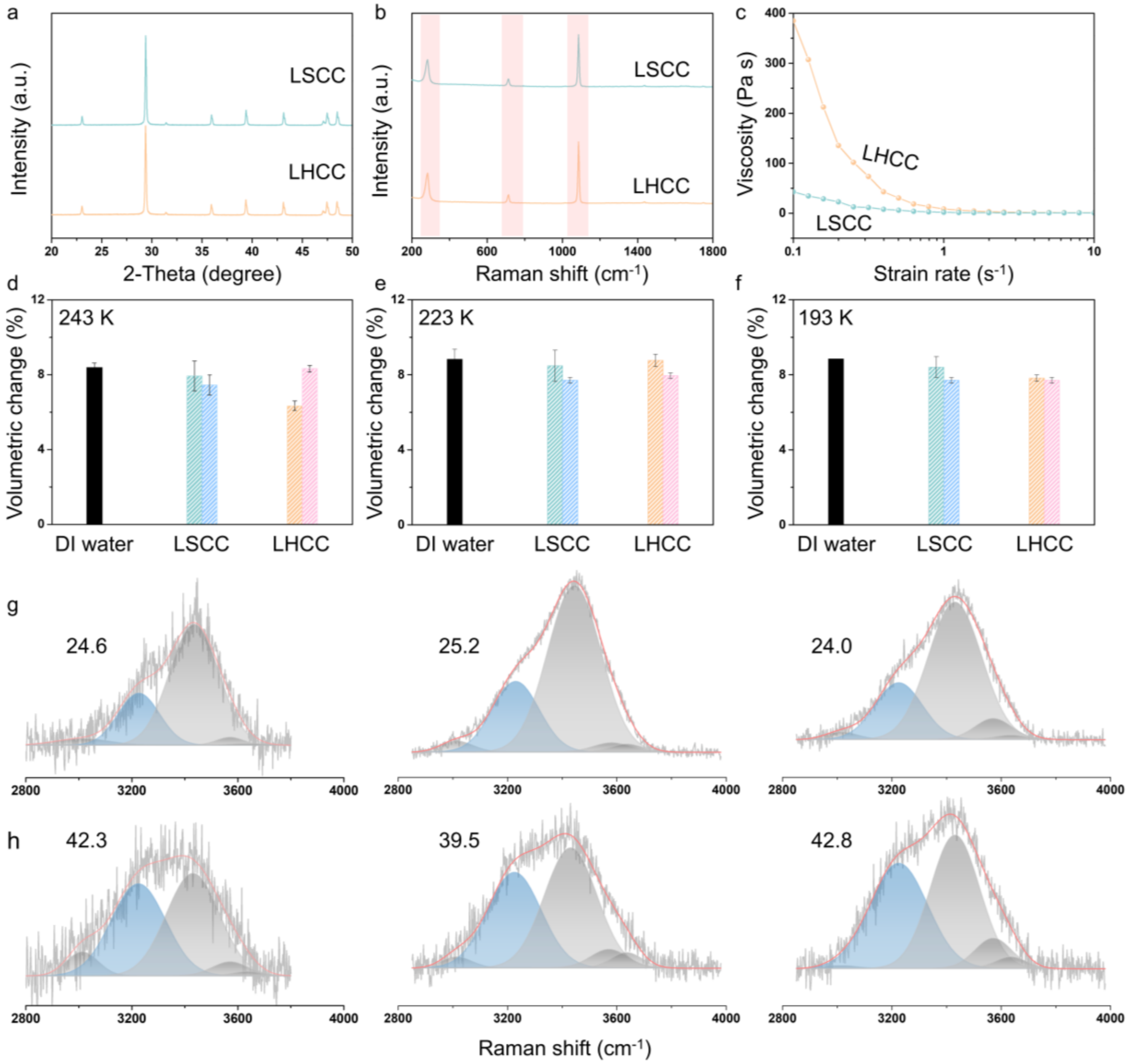

Figure S10. Structure information and the freezing behaviors of loosely packed HCC and SCC (LHCC and LSCC) (centrifuged at 2000 rpm). a) X-ray diffraction patterns; b) Raman spectra; c) viscosity; d-f) water expansion rates upon freezing at 243, 223, and 193 K for LHCC and LSCC and their corresponding supernatants. The orange and green bars stand for SCPC and HCPC, respectively. While pink and blue bars represent the corresponding supernatant. g-h) Raman spectra of LSCC(g) and LHCC(h) with fitted curves. All the spectra are activated by a laser of 532 nm and taken at ambient temperature. The inset number is the ratio of tetra-coordinated water whose sub peak is marked by blue.

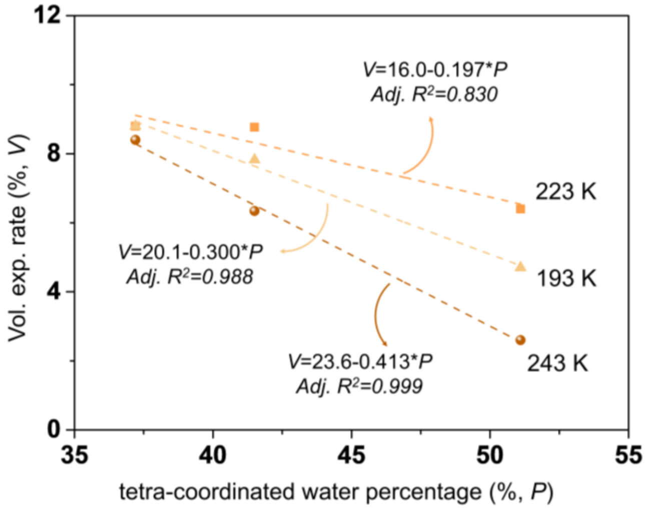

Figure S11. Linear fitting conducted on the data of DI water, LHCC, and HCC at 243, 223, and 193 K. The volumetric expansion rate is recorded by method 1 and the tetra-coordinated water percentage is the average value of three random tests.

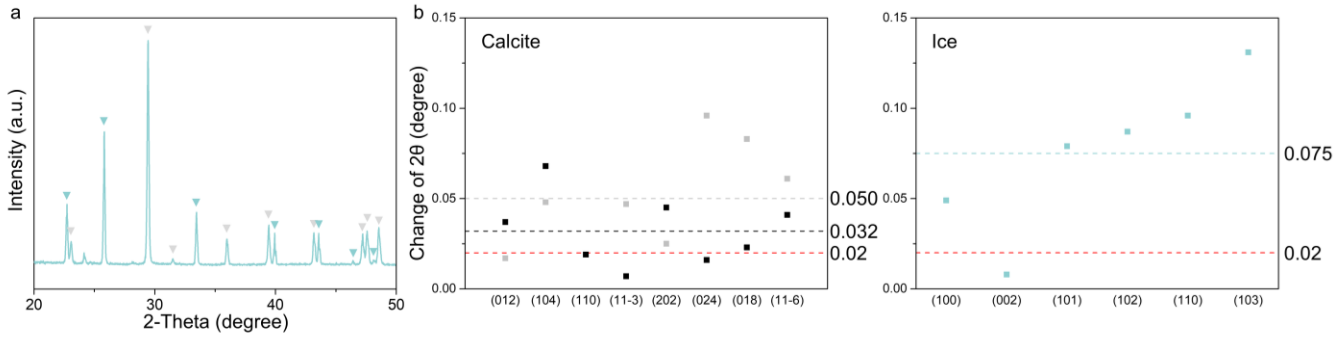

Figure S12. Investigation of XRD peaks migration of HCC at 243 K. a) XRD pattern of HCC obtained at 243 K. b) Migration level of peaks attributed to calcite and ice. The black and grey square represent the migration levels of calcite peaks obtained at room temperature and 243 K compared to standard PDF card, and the black and grey dash is the average value respectively. The cyan squares and dash line stand for the result for a single facet and their average value in ice. All the XRD patterns were measured with an interval of 0.02 degree as reflected by the red dash line.

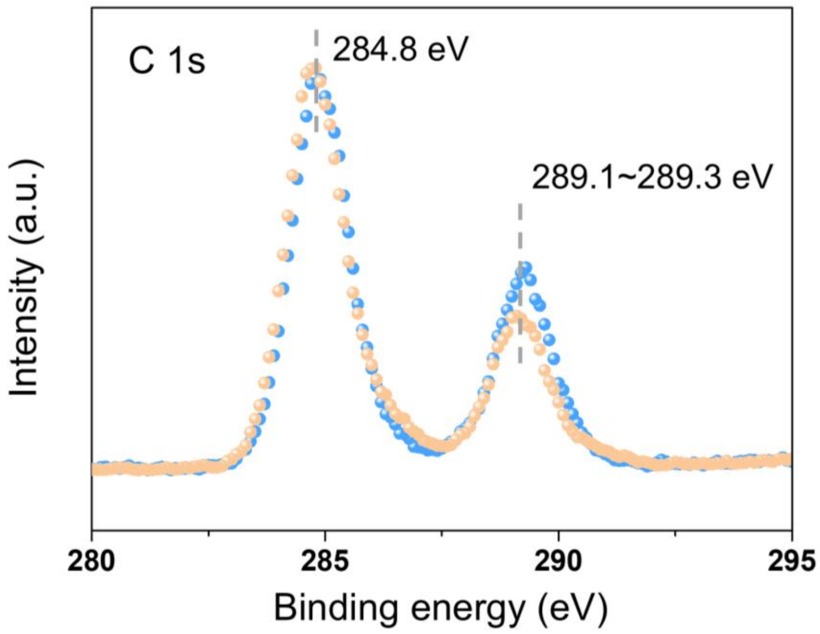

Figure S13. High-resolution C 1s spectra (XPS) of freeze-dried HCC (orange) and heat-dried HCC (blue).

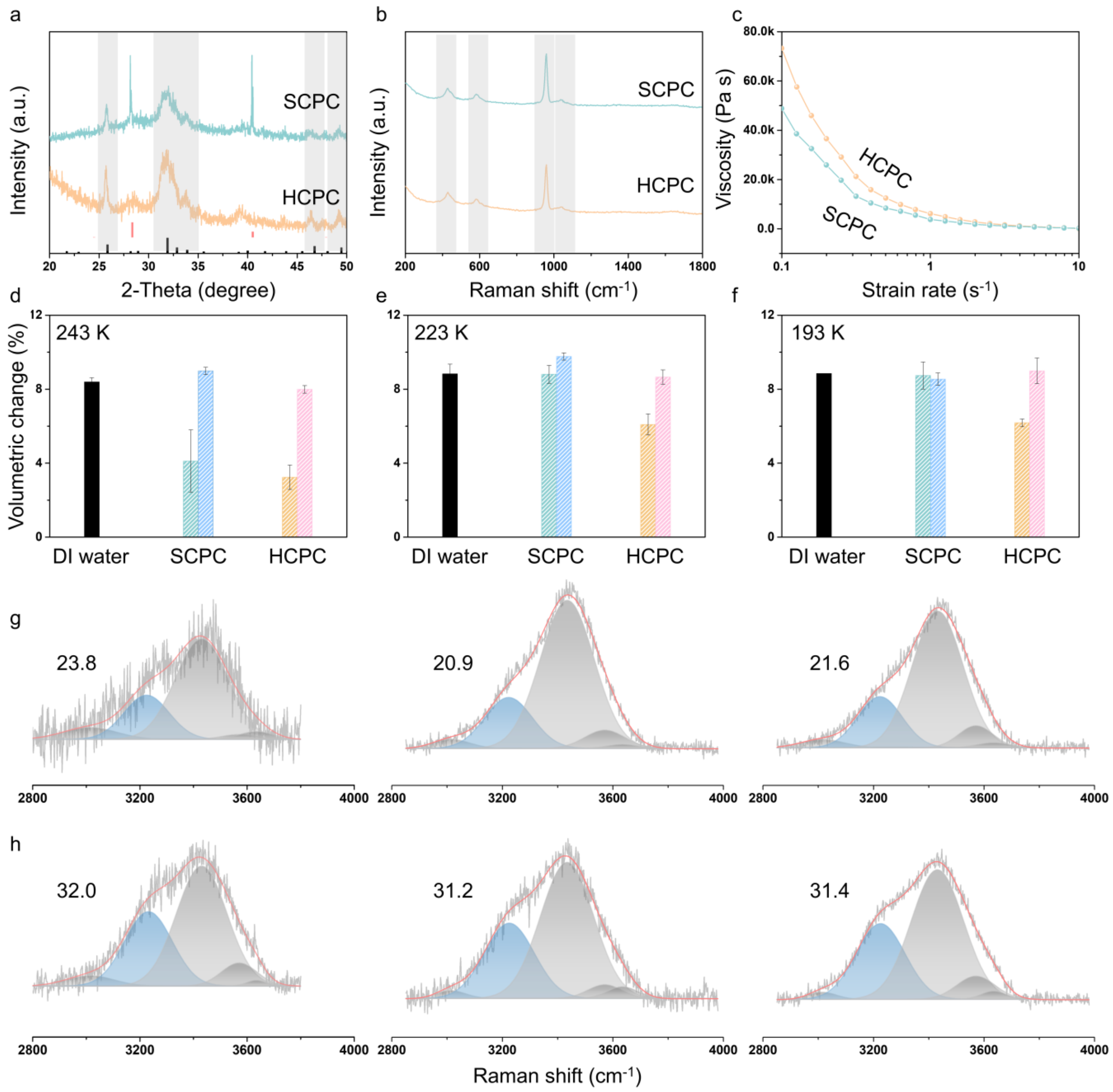

Figure S14. Structure information of tighter calcium phosphate colloids (SCPC and HCPC) and the freezing behavior. a-c) Structure of SCPC and HCPC indicated by X-ray diffraction patterns (a), Raman spectra (b), and viscosity (c). d-f) Expansion rate of tighter calcium phosphate colloids and their corresponding supernatant during freezing. The orange and green bars stand for SCPC and HCPC, respectively. While pink and blue bars represent the corresponding supernatant. g-h) Water signal obtained from Raman spectra. The inset number is the ratio of tetra-coordinated water whose sub peak is marked by blue. All the spectra are activated by a laser of 532 nm and taken at ambient temperature.

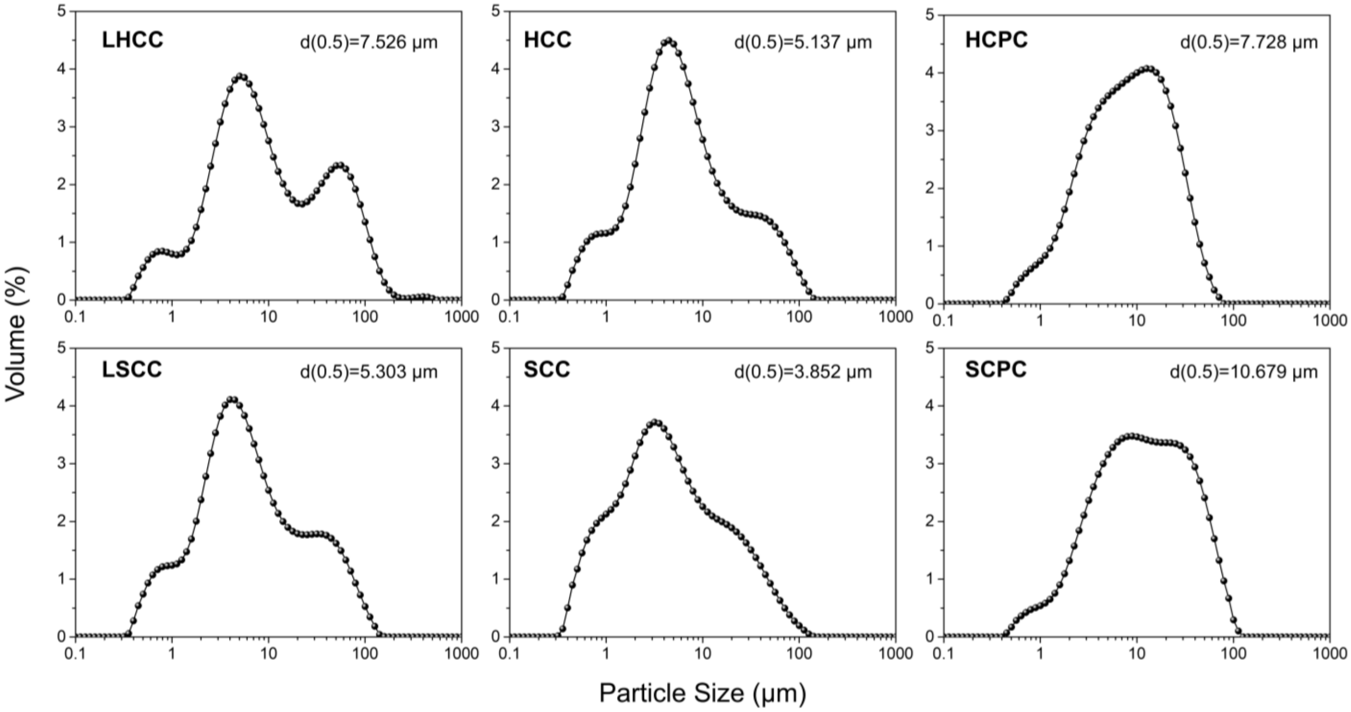

Figure S15. Particle size distribution of calcite or phosphate-based colloids after freeze-drying for 24 hours, measured using the dynamic light scattering method. The median particle size is given in the upper right corners of each graph.

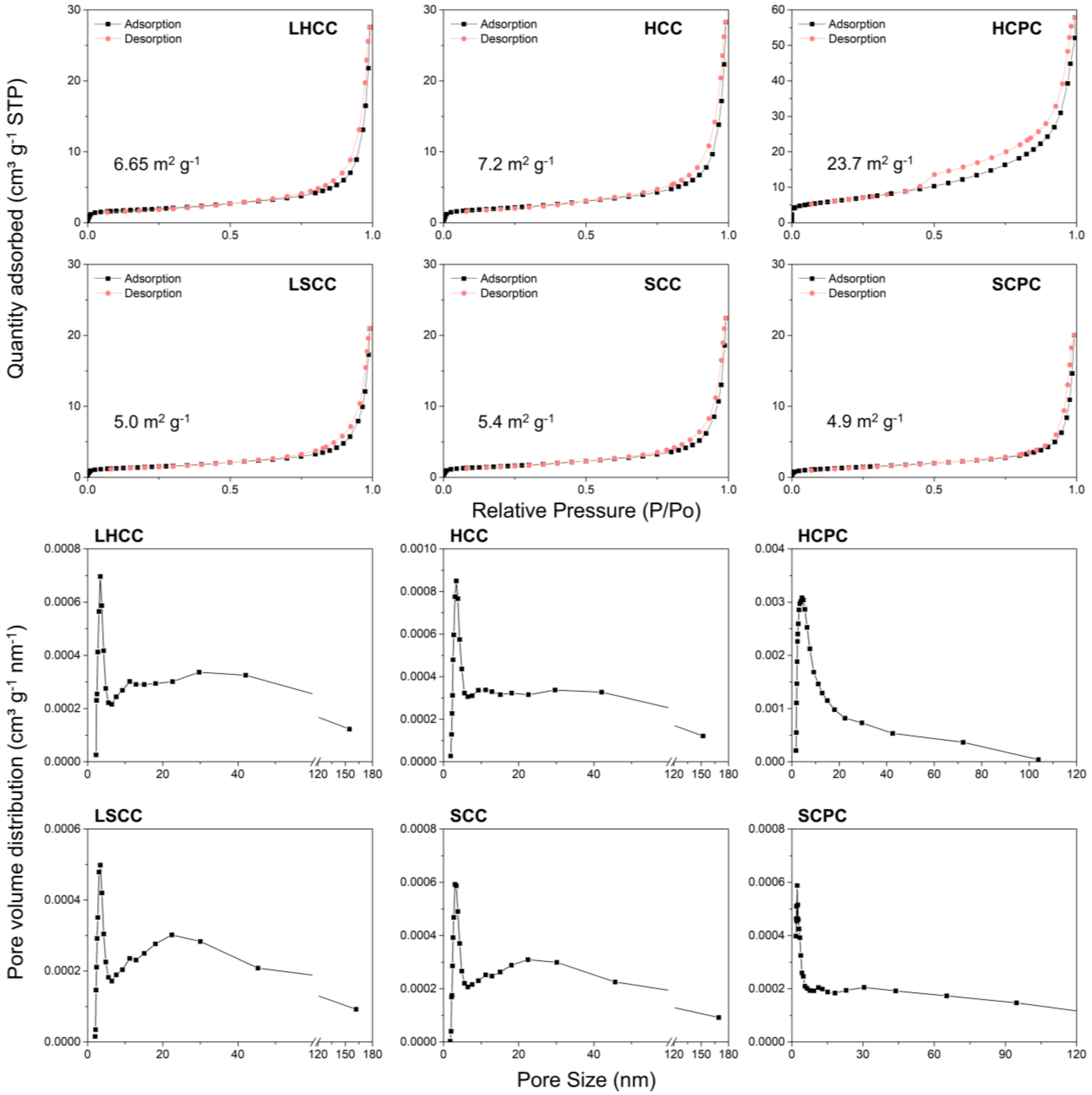

Figure S16. Nitrogen adsorption/desorption curves and pore size distribution of calcite or phosphate-based colloids after freeze-drying for 24 hours and then thermal drying at 423 K for 8 hours.

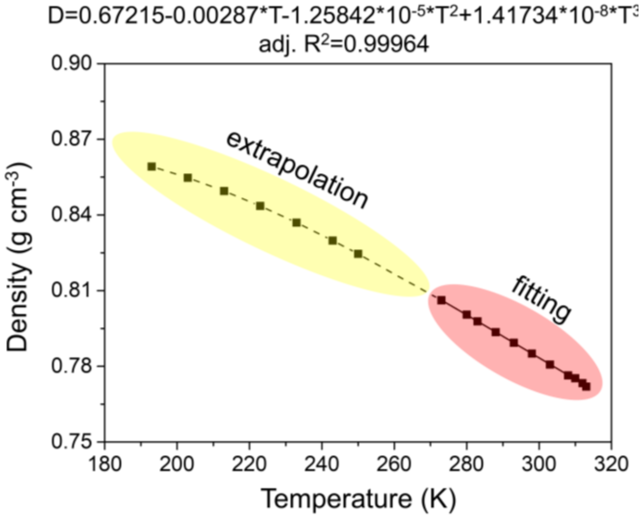

Figure S17. Fitting and extrapolation of ethanol's density, in accordance with Archimedes' principle regarding density measurement and the antifreezing properties of ethanol at low temperatures.

Table S1. Contribution coefficient of tetra-or-more-coordinated hydration layer to the reduced water expansion upon freezing. The coefficient of HCC at 223 K was only about 60%, which may be affected by the longer lengths of the hydrogen bonds at that temperature.[2]

Temp.	$R_{T,water}$ (%)	$R_{T,HCC}$ (%)	$P_{T,E,water}$(%)	$P_{C,A}$ (%)	C (%)
298 K	37.4	51.1	-	-	-
243 K	61.1	59.1	8.4	2.6	109.2
223 K	62.2	61.8	8.8	6.4	60.3
193 K	64.2	62.3	8.8	4.7	79.2

Table S2. Packing density, water content, and viscosity of calcite colloids

Sample	Density (g cm^{-3})	Water content (Vol. %)	Viscosity (Pa s)
LSCC	1.43	78.2	43
LHCC	1.50	76.5	385
SCC	1.54	72.1	1076
HCC	1.83	72.2	5215

References

1. Haynes, W. M. CRC handbook of chemistry and physics. 2014, CRC press.
2. Dean, J. A. Lange's handbook of chemistry. 1999
3. Sun, Q.. The Raman OH stretching bands of liquid water. Vibrational Spectroscopy, 2009, 51(2), 213-217.
4. Bakker, R. J. Raman spectra of fluid and crystal mixtures in the systems H2O, H2O–NaCl and H2O–MgCl2 at low temperatures: applications to fluid-inclusion research. The Canadian Mineralogist, 2004, 42(5), 1283-1314.
